# Impact of mediterranean fruit fly rearing residues and biological supplementation on performance of gimmizah chicks^☆^

**DOI:** 10.1016/j.psj.2025.105198

**Published:** 2025-04-19

**Authors:** Mahmoud H. Hatab, Nashaat S. Ibrahim, Waheed A.A. Sayed, Aml M.M. Badran, Birgit A. Rumpold

**Affiliations:** a Biological Application Department, Nuclear Research Center, Egyptian Atomic Energy Authority, P.O.13759 Egypt; b Poultry Breeding Department, Animal Production Research Institute, Agriculture Research Center, Egypt; c Department Education for Sustainable Nutrition and Food Science, Technische Universität Berlin, Marchstr.23, D-10585 Berlin, Germany

**Keywords:** Mediterranean fruit fly (*Ceratits capitate*) rearing residues, Gimmizah chicks, Biological supplementation, Performance, Biochemical indices

## Abstract

For a transformation of the global food system towards sustainability, circular approaches and nutrient-rich side-stream valorization are mandatory. Moreover, affordable and sustainable alternatives to corn, soy, and fish meal are needed in poultry production. Recently, insects and their derived products have gained research interest as alternative sources of conventional feed ingredients in poultry nutrition. The Mediterranean fruit fly (medfly; *Ceratitis capitata*) production industry using sterile insect technology amasses thousands tonnes of rearing residues annually. This study is the first to shed light on the potential use of medfly rearing residues (**MFRR**), as a partial replacement for corn and soybean in Gimmizah chicks’ diets, with or without biological supplementation (**BS**). It evaluates their effects on growth, carcass characteristics, blood indices, serum biochemical and histological changes in internal organs. A 7-week trial was conducted using 240, 15-day-old Gimmizah chicks, which were randomly divided into four groups (6 replicates, 10 birds each): the first group (T1) was fed a corn-soybean control diet, the 2nd group (T2) fed the control diet enriched with 1 ml BS/kg diet. The 3rdgroup (T3) received the control diet after replacing 10 % of corn and soybean with **MFRR** meal, while the 4th group (T4) fed the 10 % replacement by **MFRR** combined with 1 ml of BS. All groups received isoenergetic and isoprotienic diets with free access to feed and water for 49 days trial period. Compared to the control, both BS and **MFRR** inclusion with or without BS (T4 and T3, respectively) positively improved body weight, feed consumption, feed conversion, performance index and carcass yield. Blood analysis showed increased red blood cells, hemoglobin, packed cell volume, total protein, albumin, globulins, triglycerides, cholesterol, thyroxine hormone, uric acid and creatinine, with no adverse histological alteration in the bursa or intestine. In conclusion, the study suggests that MFRR can effectively replace 10 % of traditional feed ingredients, with or without BS, enhancing chicks' performance and health. Further future studies are recommended for broader application of MFRR in poultry nutrition.

## Introduction

In the poultry industry, poultry feeding is one of the key areas of poultry production since feed makes up the biggest share of the poultry production costs with approximately 60–70 percent of the total expenditure (Mallick et al., 2020). At the same time, the global market price of conventional poultry feed ingredients of both plant and animal origin (soybean, corn, fish meal, etc.) fluctuates and continues to increase. In addition to the unpredictability of feed costs for imported feed ingredients particularly soy and fish meal, they are not a sustainable option (Chobanova et al., 2023).

The increasing animal feed costs have forced many communities involved in animal husbandry to go out of business (Gwak et al., 2021; Onsongo et al., 2018). In addition, the FAO (2014) pointed out the urgent necessity to find new, easily produced and sustainable alternative feed sources for the modern poultry industry that can replace unsustainable ones as a part of a sustainable global food system. There is an urgent need for alternative sustainable feed sources with a high performance, low cost, and high availability. The use of alternative feed ingredients is expected to grow, with increasing interest in alternatives such as insects, microalgae, and underutilized side streams from food and feed production. In particular insects have already been proposed as one of the potential high-quality and sustainable sources for poultry feed (Nalunga et al., 2021; Hatab et al., 2020),

With the recent changes in the European Union legislation, the use of processed animal proteins from insects in aqua feeds was approved by EU Commission Regulation (EC) 2017/893 of May 24, 2017 (EU, 2017), and more recently the European Commission Regulation (EU) 2021/1372 of 17 August 2021, also authorized the use of processed insect proteins in poultry feed (EU, 2021). Although insects have historically usually been perceived as agriculture pests, in recent decades, the utilization of reared insect and their by-products (i.e., protein meal and fat/oil) in animal feed has garnered considerable attention and scientific interest as a novel ingredient for livestock feed, highlighted by hundreds of scientific articles that are published annually on the subject (Van Huis et al., 2021). These studies show that it is feasible to partially or fully include a variety of insect larvae or prepupae meal as well as insect fat into poultry feed as a cost-effective substitute for pricey conventional feed ingredients. This approach is currently considered as one key to satisfy an increasing global demand for sustainable and affordable animal products. Insects can be reared on by-products from the food industry or on low-value agricultural by-products and transform these low-quality substrates into valuable high-quality protein biomass, that rivals fish and soybean meals in nutritional value (Kee et al., 2023; Miranda et al., 2020). Since this approach aligns with sustainable development goals (Barragán-Fonseca et al., 2020), insect can play a significant role in a circular economy by serving as ingredient of poultry feed without competing with food production (Edea et al., 2022; Makkar et al., 2014).

The fact that chickens in the conventional poultry production system scavenge all edible materials in their environment, including insects, suggests that insects constitute a natural source of feed for poultry (Edea et al., 2022). Due to their numerous nutritional benefits such as being high-quality sources of protein, fat, minerals, essential amino acids, fatty acids, micronutrients, and vitamins (Elhassan et al., 2019; Nowak et al., 2016; Makkar et al., 2014), insects are currently one of the most promising potential poultry feed resources and are already being utilized more and more in poultry feeds around the world as a major sustainable alternative to conventional animal or plant proteins (Khayrova et al., 2024; Kierończyk et al., 2022). Furthermore, insect products are also known to contain bioactive component such chitin, lauric acid, and antimicrobial peptides that optimize animal health by modulating the animal microbiome (Hossain and Bhuiyan, 2023).

Today, insects are processed into a variety of feed products with additional nutritional properties and functional qualities, ranging from larvae meal (Chen et al., 2024; Seyedalmoosavi et al., 2022 Razmaitė et al., 2025) to various processed non defatted, partially defatted, defatted and highly defatted meals (Xin et al., 2024; Goncalves et al., 2025; Zawisza et al., 2023; Schiavone et al., 2017b, 2019), larva fat (Schiavone et al., 2017a, 2018) and insect larvae oil (Moutinho et al., 2024 Aprianto et al., 2023; Fawole et al., 2021). It was shown that insects as poultry feed had beneficial effects in terms of poultry health, growth, gut health and immune response, while no negative effects on the product quality or taste and flavor characteristics of poultry meat and eggs were observed (Dorper et al., 2024 and 2023; Nieto et al., 2023, Hatab et al., 2020).

Insect mass production for food and feed purposes is currently an emerging sector worldwide. that is expanding at an exponential rate. It is anticipated that as a result, the cost and application of insects as feed ingredients will decrease and expand (Rumpold and Schluter, 2013; Van Huis, 2020).

In the insect rearing process, in addition to larval biomass that is rich in proteins and lipids and used in animal feed and food, a considerable amount of rearing residues amasses which can constitute a significant environmental risk and that needs to be properly disposed of or valorized (Poveda, 2021).

Rearing residues – sometimes also referred to as frass - are by-products of the insect rearing process and encompass undigested rearing substrate, dead larva, unhatched eggs, excreta, and shed larval skins(Adams and Koutsos, 2024; Yildirim-Aksoy et al., 2020a, 2020b; Houben et al., 2020). Effectively managing these residues presents both an environmental burden and an opportunity for valorization in other applications. Utilizing these residues of different insect mass facilities could establish a novel approach aligned with circular bioeconomy principles, in which the insect frass from one process becomes the resource in another (Lopes et al., 2022; Slorach et al., 2019). Accordingly, this approach allows insect producers to supply poultry industries with large quantities of rearing residues as feed. Currently however, insect frass is approved for specified uses outside the feed chain and thus not approved as certified feed in the European Union (EU, 2024).

The Mediterranean fruit fly (Medfly, *Ceratitis capitata*) is one of the most damaging insect pests, causing significant economic losses in agricultural crops, and threatening food security (Enkerlin et al., 2015). It is also functioning as a model for the Sterile Insect Technique (SIT) programs, an environmentally friendly and successful approach in pest control that involves mass-producing sexually sterilized insects and then releasing them in a high number in nature to induce sterile mating in natural populations, thereby reducing their reproduction (Haytham et al., 2024; Augustinos, et al., 2021). Successful implementation of this technique requires producing a sufficient number of sterile flies. SIT programs targeting Medfly are widely used worldwide and producing e.g. 6 million sterile flies/week in South Africa (Barnes et al., 2007), 130 million sterile flies/week in Morocco (Rachid and Ahmed, 2018), and 200 million sterile flies per day in Guatemala (Dyck et al., 2021). Valorizing these large quantities of rearing residues of the SIT Medfly production into e.g. high-value feed products presents a sustainable waste management strategy.

The presence of nutrients and other beneficial ingredients in insect rearing residues suggest a possible use as feed ingredient in animal diets. The lipid component of rearing residues is primarily composed of lauric acid (Borrelli et al., 2021). Lauric acid, a unique medium chain fatty acid, is easily absorbed, provides a quick energy source upon consumption (Dayrit, 2015), has antibacterial properties and was shown to significantly improve broiler growth and immune functions (Wu et al., 2021).Additionally, insect rearing residues contains beneficial microbes and chitin, a naturally occurring biopolymer from invertebrate exoskeletons. Chitin has been shown to have a prebiotic effect on the gut microbiome, which supports healthy growth and development (Subbarayudu et al., 2020). In this respect, Adams and Koutsos (2024) reported that no differences were noted in feed intake, body weight gain or feed conversion ratio in broiler chicks fed a diet with an inclusion of 2.5, 5 or 10 % of frass of black solider fly larvae (BSFL). They indicated that BSFL frass can be an effective and safe feed ingredient option for commercial broiler production. Also, Radwan et al. (2023) included 1, 2 and 3 % of mealworm (*Tenebrio molitor*, TM) rearing residues in rabbit diets for 11 weeks and they concluded that mealworm rearing residues have the potential as feed ingredient for rabbits without unfavorable effects on growth performance and carcass traits, while improving meat quality parameters. Furthermore in aquaculture, Yildirim-Aksoy et al. (2020a, 2020b, 2023) reported that BSFL frass inclusion of up to 30 % as a replacement of fish meal in channel catfish (*Ictalurus punctatus*) or hybrid tilapia (*Oreochromis niloticus x O. mozambique*) diets, resulted in improvements in final body weight and feed intake, a better feed efficiency, a reduced mortality and resistance to diseases compared to the control diet, suggesting that the rearing residues have the possibility to enhance fish health and immune system, which may result in a decrease in antibiotic use and further increase the sustainability of aquaculture production. In addition to growth performance, trials have indicated improvements in response to disease challenge when feeding BSFL frass. In channel catfish, transcriptome and immune gene expression analyses indicated an upregulation of metabolic and immune-related genes as frass inclusion in feed increased (Sankappa et al., 2024).

On the other hand, several studies support the use of biological supplements (BS) to enhance feed utilization by birds (Elsagheer et al., 2022; Hatab et al., 2016). One of these biological supplementations used in the current study is a commercial product named ZAD probiotic, which combines digestive enzymes such as cellulase, hemicellulase, amylase, and protease with a combination of non-pathogenic, anaerobic live microorganisms (Ruminococcus Flavefaciens). This supplement has been shown to positively impact animal performance and immunity (Gado et al., 2013; Gado, 2020). It is therefore assumed that BS addition to chick diets containing medfly rearing residues (**MFRR**) meal is essential to enhancing the utilization of **MFRR** meal, particularly its content of chitin and fibrous materials, thereby maximizing nutrient digestibility and absorption. Insect chitin is a fiber; represents a significant constituent of frass and thus reduces the degree of frass digestibility in monogastric animals (Sanchez-Muros et al., 2014). This in turn affects nutrient absorption but may have a positive effect on poultry health and immunomodulatory effects (Li et al., 2019). It was therefore presumed that supplementing BS may convert the chitin into the important polysaccharide-based active ingredient ‘chitosan’ which can play a role in shaping the gut microbial community, act as an immune-stimulant, reduce the intestinal inflammation and consequently improve the growth and immune function in chicks (Wang et al., 2020).

While extensive research has been conducted on the inclusion of various insect species in poultry feed, knowledge about the suitability and possibility of using insect rearing residues, especially **MFRR**, as poultry feed ingredient is still scarce. To the best of our knowledge, no literature is available and no prior studies have yet been carried out to explore the potential use of **MFRR** in poultry nutrition nor have any similar studies been done on the potential impact of **MFRR** meal usage with or without biological supplementation (BS). This study therefore represents an important first step in evaluating the viability of **MFRR** as a poultry feed ingredient and as a partial replacement of traditional feed ingredients (corn and soybean meal) of up to 10 % and with and without the addition of BS by assessing its impacts on performance outcomes, carcass characteristics, gut health; intestinal morphology, hematological, biochemical and histological features in slow growing Gimmizah chicks during 49 day growth periods. This way it is aimed to provide new insight in the applicability of **MFRR** as a sustainable poultry feed ingredient and as a waste valorization strategy.

## Material and methods

### Location and ethics statement

The present study was performed at the experimental poultry farm of the poultry physiology and production research unit, Biological Application Department, Radioisotopes Applications Division, Nuclear Research Center, Egyptian Atomic Energy Authority, Egypt. All experimental procedures used in this research received approval from the local ethics committee of National Center of Research Radiation and Technology, Egyptian Atomic Energy Authority, under approval protocol No (REC—NCRRT 11 PA/23), ensuring humane care and protection of the animals used for experimental and other scientific purposes. In this study, all efforts were made to minimize animal suffering and were carried out in compliance with the ARRIVE guidelines.

### Source and preparation of fruit fly rearing residues

The fruit fly rearing residues (**MFRR**) were obtained from the mass rearing of Mediterranean fruit fly (medfly) *Ceratits capitate*, that were reared and maintained in the insect rearing farm of the Biological Application Department, Nuclear Research Center (**NRC**), Egyptian Atomic Energy Authority. The *medflies* were reared on a semi-artificial diet in line with the regulation for farmed insect feed consisting of the following components per 1 kg diet homogenized in blender: 283 g wheat bran as bulking agent, 70 g yeast, 130 g sucrose, 2 g sodium benzoate, 15 ml HCl and 500 ml distilled water. The *medfly* had a hatch rate of 80–85 % and a larval mortality of 5–8 %. **MFRR** were collected after the last instar larvae (third instar) pupated after seven to eight days inside the rearing trays, and consists of wheat bran, sugar residue, yeast residue, HCl, sodium benzoate and residues of larvae (excreta, dead larvae, insect skin/molting residues, un-hatched eggs). After collection, each batch of **MFRR** was oven-dried in a convection oven at 70°C for 24 h (Van Looveren et al., 2022), ground into powder using an ultra-centrifugal grinder (ZM 300 Retsch, Germany), then exposing to radiation as an effective sanitary treatment against a variety of pathogens or unfavorable pests that may infect waste, with minimal negative impact on the quality of the product (Hassan et al., 2024; Sayed et al., 2023; WHO, 1988). The irradiation process was done at dose rate of 800 Gy using a ^60^Co Gamma chamber, MC20, Russia, at the Nuclear Research Center, Egypt, with an average dose rate of 1.0 Gy *h*^−1^. The irradiated **MFRR** was stored in airtight plastic containers in refrigerator at 4°C until used to formulate the experimental diets along with other ingredients.

### Chemical analysis

Prior to the application of **MFRR** in formulating the chicken feed, proximate chemical analyses were performed in triplicate. **MFRR** were analyzed for crude protein, fiber, dry matter and ash content according to standard protocol of Association of Official Analytical Chemists (AOAC methods, 2012). Gross caloric content was determined by using a digital oxygen bomb calorimeter (Model:XRY-1A,Hunan,China). The total lipids were extracted and purified according to Folch et al. (1957). The methionine and lysine contents were analyzed using High Performance Liquid Chromatography (**HPLC**) (1260 Infinity II series, Agilent Technology, Waldbronn, Germany). Moreover, the carbohydrate content was estimated according to Albalasmeh et al. (2013). Calcium (**Ca**) and phosphorus (P) were determined by inductively coupled plasma-optical emission spectrometry (ICP-OES, Leeman Prodigy High Dispersion, USA). The results of **MFRR** chemical composition analysis and chemical composition of soybean meal and yellow corn is presented in Table (2). The nutritional value and chemical composition of corn, soybean and remaining feed components contained in experimental diets was calculated based on the chemical composition of feedstuffs according to tabular data from the Nutrient Requirements of Poultry (NRC, 1994).

### Source and composition of biological supplement

The commercial biological supplement (BS), named **ZAD**, is a combination of anaerobic bacteria and digestive enzymes (multi-mix of cellulases, hemicellulase, proteases and α-amylase). It was purchased from the Academy of Scientific Research and Technology in Cairo, Egypt, and is patented under patent number 22155 (Gado et al., 2013). The product is created in liquid form and primarily consists of a mixture of anaerobic bacteria Ruminococcus Flavefaciens 11×10^3^ CFU and digestive enzymes including 8.2 U/g cellulose, 6.2 U/g hemicellulase, 64.4 U/mg amylase, and 12.3 U/g protease. The BS supplement (1 ml/ kg diet) was dissolved in water (1:5 ratio v/v) just before supplementation to the diet at the time of feeding and the BS-water mixture (5 ml) was sprayed by a step-up procedure on 1 kg of diet, then the diet was mixed in a machine mixer to ensure complete distribution.

### Birds, housing conditions, rearing procedures and management

Before the study began, the building and all equipment was cleaned and disinfected. A total of 300 one-day-old slow growing Gimmizah chicks (local developed strain developed at the El-Gimmizah poultry research Farm, Ministry of Agriculture, Egypt) were used. It is originated by crossing of male Dokki-4 (Fayoumi × White Plymouth Rock) with female white Plymouth Rock. The chicks were housed in easy-to-clean battery brooders under controlled climate and hygienic conditions without any division or any treatment for two weeks as an adaptation and quarantine period. During this time, they were all fed a basal diet with free access to feed and water without restriction.

At 15 days of age, (average initial weight, 134.8 g) all chicks were individually weighed, and 240 healthy chicks with similar weight were chosen for the purpose of the study. These chicks were distributed equally by weight and number into 4 dietary groups, each consisting of 60 chicks, further divided in turn into 6 individual pens (cages) as replicates with 10 chicks each. The allocated groups were reared throughout the experimental period from 15 day old (experimental initiation) for 7 weeks (experimental termination) in galvanized stainless-steel, clean battery cages (1 m width × 2 m length × 0.5 m height) in the same room in an open-sided building. The cages were equipped with outdoor feeders, manually filled daily with specific mash feeds for every corresponding group and one drinking line containing the same number of automatic nipple drinker with a cup. Mash diets and fresh water were administered ad libitum and feed and water were checked to assure that each was always available. The cages were separately placed from each other by randomization for the position of the cages/ groups in the experimental room.

All chicks were kept under optimum identical and standard hygienic conditions, with the same environmental conditions until the end of trial. The environmental conditions of the room were monitored and were regularly checked. The average rearing room temperature was adjusted according to the chicks' age, starting with 30°C at the beginning of the experiment, then gradually reduced by 2°C weekly until it reached 24 °C and then kept constant. A 23-hour light and 1-hour dark cycle (L:D) was provided for the whole trial using artificial illuminating. The vaccination program was carried out according to schedule. The health status of the birds was visually inspected daily and the mortality was recorded as it occurred.

### Experimental layout and diet preparation

The experimental layout consisted of four dietary treatments, tested on Gimmizah chicks from 15 to 63 days of age (7 weeks period). The chicks were fed one of the four diets, designated as T1, T2, T3 and T4 groups. The 1st group (T1) functioned as the control group, which continued to receive the basal control diet (corn–soybean meal–based diet). The 2nd group (T2) received the basal diet supplemented with 1 ml of BS per kg diet. The 3rd group (T3) received the experimental diet in which 10 % of corn and soybean meal of basal diet was partially replaced by dried ground **MFRR** (100g/kg), and the 4th group (T4) was fed the same diet as T3 that was supplemented with 1 ml BS/ kg diet. The **MFRR** were mixed into the basal diets by a step-up procedure to ensure uniform incorporation. All experimental diets were fed in mash form, designed to be both iso-energetic and isonitrogenous for all treatments. The diets were formulated to cover the nutritional requirements of chicks following the nutritional recommendations of the NRC (1994). The nutritional value of experimental diets was calculated based on the results of our own **MFRR** analyses (Table 2) and for other feed ingredients based on the chemical composition of feedstuffs according to tabular data of the NRC (1994). The detailed dietary treatments, diets composition and nutritional value are provided in Table 1.

**Table 1:** Ingredient composition and calculated chemical analysis of the experimental diets.

Ingredients %	Experimental diets			
	T1	T2	T3	T4
Yellow corn	66.25	66.25	61.70	61.70
Soybean meal (44 %)	29.2	29.2	24.2	24.2
**MFRR**	0	0	10	10
DL-methionine	0.25	0.25	0.2	0.2
Choline chloride	0.25	0.25	0.25	0.25
L-Lysine	0.15	0.15	0.2	0.2
Dicalcium phosphate	2	2	1.3	1.3
Limestone	1.3	1.3	1.55	1.55
Sodium chloride	0.3	0.3	0.3	0.3
Vitamin and mineral premix^1^	0.3	0.3	0.3	0.3
Calculated chemical analysis				
Crude protein	19.07	19.07	19.11	19.11
Crude fiber	3.5	3.5	4.0	4.0
Lysine	1.1	1.1	1.1	1.1
Methionine	0.55	0.55	0.56	0.56
Methionine + cysteine %	0.6	0.6	0.76	0.76
Calcium	1.03	1.03	1.15	1.16
available phosphorus	0.51	0.51	0.58	0.0.58
Metabolizable Energy MJ/kg	12.04	12.08	12	12

1 vitamin-mineral premix provided per kg diet: IU: vit. A 4,000,000, vit. D3 500,000; g: vit. E 16.7, vit. K 0.67, vit. B1 0.67, vit. B2 2, vit. B6 67, vit. B12 0.004, nicotinic acid 16.7, pantothenic acid 6.67, biotin 0.07, folic acid 1.67, choline chloride 400, Zn 23.3, Mn 10, Fe 25, Cu 1.67, I 0.25,Se 0.033,Mg 133.4.

### Growth performance parameters

During the 49-day experimental period, growth performance parameters were assessed weekly on pen basis using a high precision electronic scale (KERN PLE-N v. 2.2; KERN & Sohn GmbH; d: 0.1 g). At the start of the experiment and before being placed the chicks into the cages bird’s, initial body weight was recorded to the nearest gram and then weekly thereafter on individual basis to evaluate the average weekly live body weight (**LBW**). Feed consumption (**FC**) was also recorded weekly by subtracting the remaining feed in the feeder from the amount given, subsequently the total consumption was calculated on a cumulative basis. Total weight gain and total feed intake were used to calculate the feed conversion ratio (**FCR**) at the end of experiment, defined as the ratio of the amount of feed consumed throughout the experimental period divided by the body weight gained during that period (gm feed /gm body weight). Mortality was monitored daily, recorded as it occurred and deceased chicks' weights were factored into FC and FCR calculations. Weekly data on body weight and feed consumption were documented to calculate average daily feed intake (**ADFI**) and average daily weight gain (**ADG**). Additionally, the performance index (**PI%**) were calculated according to Abdel Moati et al. (2022).

### Slaughtering Performance

At the end of the experimental period, all birds were weighed individually to determine the average body weight of each group. From each replicate, three birds (18 birds/group) were selected, based on the average weight to avoid bias effect by selecting chicks with very low or very high body weights. Selected birds were off feed for 6 h with free access to water, then individually weighed, slaughtered under sterile conditions to full bleeding with stress-reducing techniques such as gentle handling and limiting handling time. Blood samples were collected during bleeding. After complete bleeding and removal of feathers, head, and legs, the skin was cut off and all the internal organs were extracted to obtain an eviscerated carcass. The eviscerated carcasses, internal viscera (liver, kidney, gizzard, proventriculus, heart, spleen, Bursa and intestine) and abdominal fat were weighed individually, calculated and represented as a percentage of fasted live body weight. All measurements were taken using a calibrated digital scale to ensure accuracy. Besides, the caecum was also isolated and photographed orthogonally, the images were subsequently analyzed with the Image®-Pro Plus software (6.0 version, Media Cybernetics) in order to record the caecal length. Furthermore, Bursa and intestine samples were collected for a histological study.

### Blood sampling, hematological and serum biochemical evaluation

Hematological and biochemical parameters were evaluated to gather information about the chicks’ health profiles. During slaughtering and bleeding, individual blood samples were collected from the 18 selected birds from each treatment group. The sample per bird was split into two equivalent portions and transferred in 2 sterile clean tubes, one with heparin as an anticoagulant for hematological analysis and one without heparin for biochemical analysis. For hematological analysis, hemoglobin concentration (**HB%**), packed cell volume percentage (**PCV%**) (Dacie and Lewis, 1991), and total red blood cells (**RBCs, 10^6^ cell/μL**) counts (Toghyani et al., 2010) were measured using an automatic blood analyzer (ADVIA 120 Siemens, Munich, Germany). For biochemical analysis, the second blood sample (non-heparinized samples) was left at room temperature in a standing position for approximately 2 h to favor blood clot, then centrifuged at 4000 rpm for 10 min to separate the serum. The resulting clear serum was carefully harvested, sampled to new Eppendorf vials, subsequently divided into aliquots and stored at −20°C to avoid multiple freeze-thaw cycles and maintain sample integrity until the time to perform subsequent hormonal and biochemical analysis. The serum was analyzed for protein profile (total proteins; **TP,** albumin; **Alb,** and globulins; **Glob**; difference between albumin value from their corresponding total protein value of the same sample); liver function (alanine aminotransferase; **ALT**, aspartate aminotransferase; **AST,** and alkaline phosphatase; **ALP**), kidney function (urea and creatinine; **CRE**) and lipid profile (cholesterol; **Chol** and triglycerides; **TG**) using spectrophotometric technique with a compact clinical chemistry analyzer system (ABX Pentra 400, Horiba-ABX, Montpellier, France) with commercial available calorimetric respective kits provided by Biodiagonistic (Biodiagnostic company, Egypt) following the manufacturer's guidelines. Furthermore, serum total thyroxine (**T4**) hormone concentration was determined by Radioimmunoassay (RIA) technique using solid phase coated tubes and tracer labeled with I^125^ using available respective detection kits (ISOTOPES Co Ltd, Budapest, Hungary) and the samples were counted on a Packard Cobra Gamma Counter (Perkin-Elmer Life Sciences Inc., Boston, MA). according to the procedures provided by the manufacturers.

### Histological observations

Tissue samples of Bursa and small intestine were excised and collected immediately from the same slaughtered birds of each groups and processed for histological structure studies following the procedures previously described (Bancroft and Layton, 2019). The small intestine was flushed with 0.9 % saline to remove all content until no digesta content was visible. Briefly, both tissues (Bursa and small intestine) were cut into 3-4 mm slices, fixed in 10 % neutral buffered formalin for 24 h, after which their solution was changed and kept in formalin buffer until testing. Tissues were dehydrated in graded serial concentrations of ethanol, cleared in xylene and embedded in paraffin wax blocks. The paraffin blocks were sectioned with a microtome at (4-6 μm) thickness, mounted on glass microscopic slides, dyed with Hematoxylin and Eosin (**HE**) stain and the stained slides were examined under a Leica microscope (CH9435 Hee56rbrugg, Leica Microsystem, Switzerland)

### Statistical analysis

The collected data were statistically analyzed by one-way analysis of variance (ANOVA) as a completely randomized design using the General Linear Model (**GLM**) procedure under statistical analysis system software SAS 9.4 (SAS Institute Inc, 2015).Data are presented as means ± standard error (SEM) of mean. Duncan multiple range test (Duncan, 1955) was applied to identify significant differences between treatment means and were defined to be statistically significant at *P* ≤ 0.05.. The statistical model used was : Yij = μ + Ti + eij, where Yij is the observation, μ = the overall mean, Ti = the fixed treatment effect (*i* = 1, 2, 3, and 4; where 1= control, 2=treatment 1, 3=treatment 2, and 4 = treatment 3) and eij = random error.

## Results

### Nutritive value of MFRR

The proximate compositions of yellow corn, soybean meal, and **MFRR** are presented in Table 2. A reasonable level of crude protein of 26.95 % was observed in **MFRR**, which indicates its applicability for poultry feed. Along with proteins, **MFRR** is also a very good source of lipids with a lipid content of 7.12 % compared to 1.9 % and 4 % for soybean and corn respectively. Moreover, **MFRR** meal had higher fiber, methionine, calcium and phosphorous contents than both corn or soybean. The lysine content in **MFRR** was higher than in yellow corn but lower than in soybean meal. **MFRR** also contained 47.1 % of carbohydrates, which was higher than soybean meal (32.5 %) but lower than yellow corn (70.0 %).

**Table 2:** Average nutrient compositions of **MFRR**, yellow corn and soybean meal in *%* (based on dry matter).

**Nutrient**	**MFRR meal**	**Soybean meal**	**Yellow corn**
Dry matter	91.8	88	88
protein	26.95	44.0	8.5
Total lipid	7.12	1.9	4
Fiber	8.9	7.0	2.5
Carbohydrates	47.1	32.5	70.0
Calcium	1.8	0.29	0.02
Total Phosphorus	2.4	0.65	0.28
Lysine	1.0	2.69	0.26
Methionine	0.97	0.62	0.18
Metabolizable Energy (kcal/kg**)**	2361	2230	3350

### Productive performance of gimmizah chicks

The health status of chicks in all groups was consistently good throughout the study. All birds remained healthy, with no signs of illness, respiratory or gastrointestinal issue and a zero mortality rate. The overall growth performance of Gimmizah chicks as affected by dietary treatment are detailed in Table 3, highlighting the beneficial effects of BS supplementation and partially replacement of corn and soybean with 10 % **MFRR** with or without BS. At the start of the study, the average initial live body weight was approximately the same for all groups. But with each passing experimental week, the experimental diets significantly affected live body weight (LBW) of chicks. The average body weight of live chicks recorded in T2, T3 and T4 was significantly higher (*P* ≤ 0.05) than that of the control group (T1). These results suggested that both BS (T2) and **MFRR** inclusion with and without BS (T4 and T3 respectively) are effective ingredient in chicks feed, positively impact the live body weight, specifically, T4-fed chicks exhibited the greatest weight gain (*P* ≤ 0.05) in comparison to all other dietary groups.

**Table 3:** Effects of biological supplementation (BS) and **MFRR** inclusion with/without biological supplementation (BS) on productive performance of Gimmizah chicks.

Item	Experimental groups			
	T1	T2	T3	T4
**Live Body Weight (g)**				
**Initial weight**	**134.81±3.27**	**134±3.85**	**134.69±3.25**	**135.86±3.92**
**Initial weight**	134.81±3.3	134±3.85	134.69±3.25	135.86±3.92
**1st week**	203.83^b^±5.6	204.83^b^±5.5	242.95^a^±26	251.58^a^±7.05
**2nd week**	274.08^c^±6.96	297.95^b^±7.4	315.85^a^±5.05	324.08^a^±8.81
**3rd week**	314.26^c^±6.2	330.04^bc^±7.9	349.47^ab^±7.6	356.3^a^±7.74
**4th week**	488.7^d^±7.6	505^c^±4.3	514.6^b^±4.90	525.6^a^±5.22
**5th week**	560.4^c^±10.7	646.4^b^±9.14	658.5^b^±9.23	686.1^a^±11.25
**6th week**	695.4^c^±10.2	750.1^b^±7.8	730.6^b^±7.15	808.2^a^±8.4
**7th week**	796.66^c^±5.9	870^b^±4.6	880.06^b^±4.62	940.3^a^±5.95
Feed consumption (FC); (g/bird/day)				
**1st week**	19.06^a^±0.3	18.9^a^±0.4	18^b^±0.4	18.2^b^±0.5
**2nd week**	23.8^b^±0.5	22.9^b^±0.2	27.08^a^±0.2	27.48^a^±0.3
**3rd week**	28.27^c^±0.15	29.28^b^±0.5	32.35^a^±1.6	33^a^±1.7
**4th week**	33.7^b^±1.3	34.8^a^±1.9	36.9^a^±2.2	37.5^a^±2.5
**5th week**	36.05^c^±1.4	37.5^b^±1.4	39.1^a^±1.5	40.1^a^±1.5
**6th week**	41.7^b^±1.3	42.8^b^±1.9	43.9^a^±2.2	45.5^a^±2.5
**7th week**	44.5^b^±1.2	45.5^b^±1.3	47.0^a^±1.3	48.05^a^±1.5
Feed Performance				
**Cumulative feed consumption (g/bird)**	1589^d^	1622^c^	1710.3^b^	1748.8^a^
**Feed conversion ratio (FCR)(feed: gain)**	2.4^a^	2.17^b^	2.32^a^	2.17^b^
**Average daily weight gain (ADG, g)**	13.51	15.2	15.00	16.42
**Total average weight gain (g/bird)**	661.85	736	745.37	804.44
**Average daily feed intake (ADFI) (g/bird)**	32.44	33.1	34.9	35.7
**Performance Index (PI %)**	331.9	401	379.34	433.32

Values are mean± SEM.

a, b, c Means in the same row with different superscripts are significantly different at (*P* ≤ 0.05). Performance index (PI) = live body weight in g / FCR×100.

The same trend was also observed in feed consumption. Feed consumption was also higher in the treated groups, with cumulative feed consumption of 1,589, 1,622, 1,710, and 1,749 g/ bird for T1, T2, T3, and T4, respectively. Furthermore, dietary treatments exerted a significant effect in improving FCR. Treated groups requiring less feed per unit of weight gain compared to the control group. Essentially, the treated groups all showed an improved feed conversion efficiency of 2.17 for T2, 2.32 for T3, and 2.17 for T4 when compared to 2.4 for T1. These results suggested that including **MFRR** in the chicks' diet, especially when combined with BS could be adequately used as an ingredient for chicks feed to get higher body weight, higher feed consumption and better feed conversion efficiency in comparison to commonly used traditional commercial feeds.

### Slaughtering performance

Table 4 displays the carcass characteristics and relative internal organs weights in Gimmizah chicks as affected by dietary treatments. Statistical analysis (*P* ≤ 0.05) revealed significant differences in carcass weight among the groups. Treated chicks (T2, T3 and T4) had a significant higher carcass yield compared to the control, with T4 was superior in this concern, showing the highest carcass weight. Results also showed that partially replacing corn and soybean meal with **MFRR** with or without BS (T4 and T3, respectively), significantly (*P* ≤ 0.05) increased the relative liver, heart, gizzard, spleen, bursal weights and cecum length compared to the control group (T1) and BS group (T2). No significant differences were observed between T2 and control regarding these organs weights. Conversely, the relative weight of the intestine was significantly lower in treated groups compared with the control group, with the most pronounced reduction in the T4 group. No significant differences were observed in the relative weight of the kidney, proventriculus and abdominal fat among all four experimental groups.

**Table 4:** Effects of biological supplementation (BS) and **MFRR** inclusion with/without biological supplementation (BS) on carcass and internal organs’ relative weights (gm/100 gm) of Gimmizah chicks.

item	Experimental groups			
	T1	T2	T3	T4
**Carcass %**	56.0^c^±2.78	58.33^b^±1.78	59.0^ab^±1.79	61.23^a^ ±1.63
**Liver %**	2.276^b^±0.02	2.313^b^±0.023	2.45^a^ ±0.01	2.48^a^ ±0.015
**Heart %**	0.466^b^±0.02	0.473^b^±0.02	0.566^a^ ±0.014	0.6^a^ ±0.03
**Proventriculus%**	0.362±0.016	0.402±0.03	0.435 ± 0.03	0.416 ± 0.02
**Gizzard%**	2.613^c^±0.14	2.602^c^±0.16	2.761^b^ ±0.132	3.013^a^ ±0.128
**Spleen %**	0.0881^c^±0.007	0.109^b^±0.0034	0.127^a^ ±0.0045	0.131^a^ ±0.0036
**Bursa %**	0.216^b^±0.014	0.221^b^±0.0069	0.249^a^ ±0.0074	0.256^a^ ±0.0055
**Kidney%**	0.608±0.029	0.0588±0.033	0.0621±0.035	0.614±0.032
**Intestine%**	15.69^a^±0.25	14.52^b^±0.06	14.41^b^ ±0.03	13.43^c^ ±0.29
**Cecum length (cm)**	11.76^b^±0.12	11.83^b^±0.09	12.35^a^ ±0.16	12.46^a^ ±0.16
**Abdominal fat%**	0.81±0.03	0.806±0.022	0.83±0.013	0.84±0.01

Values are mean± SEM.

a, b, c -Means in the same row with different superscripts are significantly different at (*P* ≤ 0.05).

### Hematological and biochemical constituents´ response

Table 5 lists the influences of dietary BS and partial substitution of corn and soybean with **MFRR** with or without BS on blood hematology and serum biochemical indices in Gimmizah chicks**.** Results revealed that all hematological measures, hemoglobin (Hb%), packed cell volume (PCV%) and red blood cell counts (RBCs) were significantly increased in treated groups as compared with control group and no significant differences were found between treated groups themselves. Serum analysis revealed that serum content of total proteins (TP), albumin (Alb), globulins (Glob), triglycerides (TG), cholesterol (Chol), urea, creatinine (Cre) and thyroxine hormone were significantly increased in the three treated groups (T2, T3 and T4) relative to the control group (T1). The T4 group showed the highest values and significantly surpassed all other groups, while T2 and T3 did not differ significantly from each other. However, no significant differences were observed among values in serum concentration of hepatic function biomarkers (ALT, AST, ALP) when comparing all experimental groups.

**Table 5:** Effects of biological supplementation (BS) and dietary **MFRR** inclusion with/without BS on blood indices and serum biochemical measurements of Gimmizah chicks.

Parameters	Experimental groups			
	T1	T2	T3	T4
**RBCs (10^6^×mm^3^)**	3.886^b^ ±0.23	4.441^a^ ±0.19	4.345^a^ ±0.2	4.494^a^ ±0.17
**Hb (g/dl)**	12.76^b^ ±0.33	13.4^a^ ±0.28	13.4^a^ ±0.3	13.6^a^ ±0.19
**PCV%**	32.3^b^ ±1.11	33.6^a^ ±1.17	33.6^a^ ±1.08	33.9^a^ ±0.93
**TP (g/dl)**	4.1^c^ ± 0.03	4.4^b^ ±0.02	4.3^b^ ±0.04	4.6^a^ ±0.04
**Alb (g/dl)**	2.4^c^ ±0.01	2.6^b^ ±0.01	2.5^b^ ±0.03	2.7^a^ ±0.02
**Glob (g/dl)**	1.7^c^ ±0.02	1.8^b^ ±0.01	1.8^b^ ±0.02	1.9^a^ ±0.02
**ALP (IU/L)**	207.3 ± 4.0	207.6 ± 4.8	208.7 ± 4.3	207.0 ± 5.5
**ALT (U/ml)**	30.392 ± 2.33	30.149 ± 1.91	30.427 ± 2.24	30.635±1.68
**AST (U/ml)**	36.122±1.68	36.414 ± 1.85	36.718 ± 1.79	36.154 ± 1.5
**TG (mg/dl)**	147.2^c^ ±2.01	159^b^ ±1.86	161.2^b^ ±2.19	165.1^a^ ±2.0
**Chol (mg/dl)**	186.6^c^ ±2.6	192.2^b^ ±2.41	190.8^b^ ±3.1	197.1^a^ ±1.91
**Urea (mg/dl)**	25.33^b^ ±0.88	27.4^a^ ±0.54	28.16^a^ ±0.88	28.1^a^ ±0.92
**Cre (mg/dl)**	0.5^b^ ±0.05	0.63^a^ ±0.04	0.65^a^±0.03	0.72^a^ ±0.05
**Thyroxine (T4) (nmol/L)**	58^c^ ± 0.88	60.9^b^ ±0.85	60.5^b^ ±0.75	63.15^a^ ± 0.9

Values are mean± SEM.

a, b, c -Means in the same row with different superscripts are significantly different at (*P* ≤ 0.05). RBCs red blood cell counts, Hb hemoglobin, PCV packed cell volume, TP total proteins, Alb albumin, Glob globulin, ALP, alkaline phosphatase, ALT alanine aminotransferase, AST aspartate aminotransferase, TG triglycerides, Chol cholesterol, Cre creatinine.

### Histological investigation

**Fig. 1:**
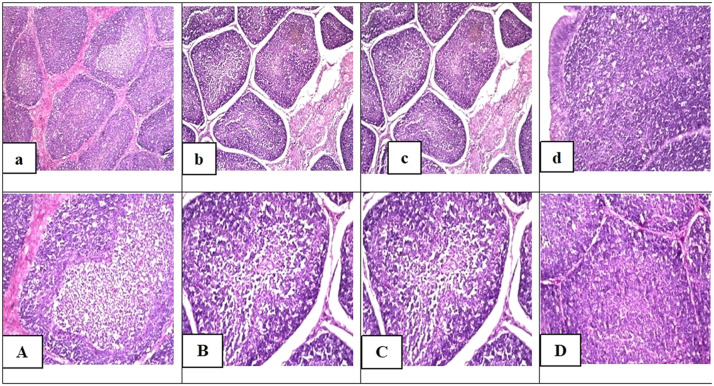
Photomicrographs presenting the effect of dietary BS supplementation and partial replacement of corn and soybean meal with **MFRR** in the presence or absence of BS on histological alterations in the bursa of tested chicks. The Low and high magnification of sections of Bursa of the control group (**a,** and its magnification **A**) showing depletion in the central portion of some individual lymphoid follicles. Histological study for the sections of Bursa of fabricus of the T2 (**b,** and its magnification **B**), T3 (**c,** and its magnification **C**) and T4 groups **(d,** and its magnification **D)** showing normal histological structure.(Photos of a, b, c and d: magnification power = *X* 160. Photos of A,B,C and D: magnification power = *X* 400).

Histological findings in the bursal sections of birds that received dietary BS (T2) and **MFRR** inclusion with and without BS (T4 and T3 respectively) revealed normal structure and no histopathological alteration or any appreciable changes were observed (Fig. 1; b, c and d respectively). However, microscopical examination in the bursa of the control group (Fig. 1a), showed histological changes as seen by a in depletion in the central portion of some individual lymphoid follicles.

**Fig. 2:**
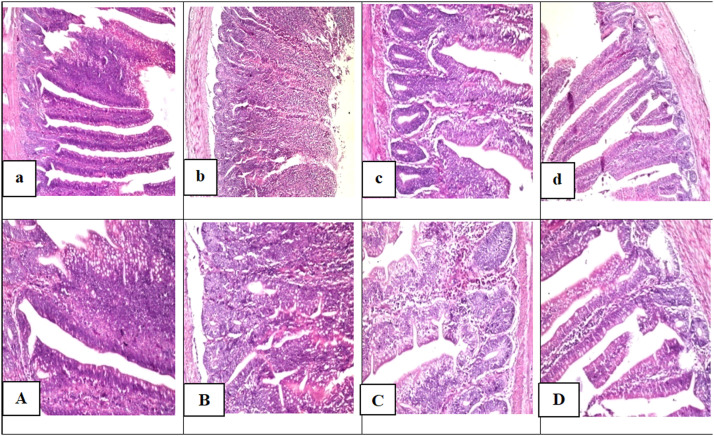
Photomicrographs exhibiting the effect of partial replacement of corn and soybean with **MFRR** in the presence or absence of BS supplement on histological alterations in the small intestine of tested chicks . Histological finding in the sections of control group **(T1) (a,** and its magnification **A)** and BS group **(T2)** (**b,** and its magnification **B**), showing fusion of the villi with absence of the crypts.. Small intestine of **MFRR** meal inclusion (T3) (**c,** and its magnification **C**) and **MFRR** meal inclusion +BS (T4) (**d,** and its magnification **D**) showing normal histological structure with wide and tall crypts between the villi. .(Photos of a, b, c and d: magnification power = *X* 160. Photos of A,B,C and D: magnification power = *X* 400).

Histological examinations of the small intestine segments of Gimmizah chicks fed **MFRR** meal (T3) and **MFRR** supplemented with BS (T4) displayed normal histological structure of the villi with wide and tall crypts in between the villi (Fig. 2;c and d, respectively). On the other hand, histological changes were observed in the small intestine of birds fed both the control diet (T1) and the control diet supplemented with BS (T2) as shown by fused villi with the absence of crypts (Fig. 2; a and b, respectively).

### Disscussion

This study explores the viability and possibility utilization of using rearing residues obtained from the biomass of Mediterranean fruit fly (Medfly) *Ceratits capitate* as a partial replacement for soybean and corn in Gimmizah chick diets with and without biological supplementation (BS). It documents the effects of this substitution over a 49-day growing period on productive performance, carcass characteristics, blood hematological, biochemical, and histological change. The aim is to provide new insights and knowledge into the targeted applicability of these rearing residues as alternative and sustainable feed ingredients.

In the current study, all experimental diets contained the same ingredients (except for BS and **MFRR**) and were designed to be isoenergetic and isonitrogenous, to eliminate any potential effects of different energy and protein levels on the results. According to our results, throughout the trial all birds remained healthy with no signs of illness and a zero mortality rate in all groups, indicating no adverse health effects**.** This finding is consistent with several studies that advocate and supports the inclusion of insects or their derivatives in poultry diets, showing that such diets are highly interesting and were greatly appreciated by the poultry species, and no mortality was recorded (Vasilopoulos et al., 2023; Nieto et al., 2022; Dzepe et al., 2021). Since in nature, insects are part of poultry diets, chickens that have access to outdoor zone pick up and eat them on a voluntary basis at all life stages. This behavior suggests that they are evolutionarily adapted to insects as a natural part of their diet (Oddon et al., 2021; Biasato et al., 2018a).

Obtained results showed that supplemented Gimmizah chicks diet with BS and inclusion of 10 % **MFRR** with or without BS as a partial replacement of corn and soybean resulted in significantly positive effects with regard to productive performance. Using this approach, not only corn and soybean meal could be substituted with an organic side-stream, but **MFRR** also possess additional functional benefits, that led to a significant increase (*P* ≤ 0.05) in live body weight, feed intake and the best feed conversion rate (Table 3) in comparison to chicks that were fed control diets containing conventional feed ingredients.

The findings of the current study align with previous research on the use of frass (insect rearing residues) from black solider fly larvae (BSFL) as feed ingredient. Adams and Koutsos(2024) determined that frass from BSFL can be used as a sustainable, effective and safe feed ingredient for broilers. They observed no differences in feed intake, body weight gain or feed conversion ratio when BSF frass was included at levels of 2.5 %, 5.0 % and 10 % in the broiler diet. Similarly in aquaculture, Yildirim-Aksoy et al. (2020a, 2020b) tested BSFL frass at varying percentages (5, 10, 20, and 30 %) as feed ingredient as a partial replacement of soybean meal and corn in Channel Catfish and Hybrid Tilapia, respectively, demonstrating that BSFL frass improved growth, hematological, biochemical parameters, disease resistance, and reduced mortality. This suggested that the frass can enhance the sustainability of aquaculture production. In addition to growth performance, Sankappa et al. (2024) found that BSFL frass inclusion upregulated metabolic and immune genes expression in Channel Catfish. However, Radwan et al. (2023) reported that replacing wheat bran and soybean meal with 1, 2, or 3 % of mealworm *(Tenebrio molitor*) frass in diets of Gabali rabbits for 11 weeks had no significant effects on growth, carcass traits, or blood biochemistry, indicating no adverse health effects from insect frass and its feasibility of wheat bran and soybean meal replacement in rabbit feed.

The enhanced productive performance observed in the present study could be attributed to a number of aspects. Primarily, it is due to increased feed intake, as the tested diets had similar metabolizable energy, protein content, and particle size, and all chicks were raised under the same consistent environmental conditions. The higher feed intake in chicks fed diets containing 10 % **MFRR**, with or without BS, could be linked to the higher fat content (Table 2), which could increase the palatability of these diets. This is supported by findings from (Yildirim-Aksoy et al., 2020a, 2020b) and Al-Qazzaz et al. (2021) who associated improved growth performance with better fat digestibility in broilers fed replacement diets.

Additionally, insect meal has been shown to be resemble animal–based proteins than plant origin protein (Hong et al., 2020). Animal protein sources have higher bioavailability and a rich amino acid profile, particularly lysine and methionine (de Souza-Vilela et al., 2019; Dzepe et al., 2019). Insects provide bioactive compounds, such as phenolic compounds and flavonoids, which act as antioxidants, anti-inflammatory agents, and antimicrobials (Nino et al., 2021; Da Silva-Lucas et al., 2020), reducing the antibiotics use in the poultry industry, controlling the antimicrobial resistance and its adverse effects on human health (van Huis, 2013). Besides its promising value as a feed ingredient, insects and their byproducts also contain beneficial compounds such as chitin and lauric acid, which may contribute to reduce harmful bacteria and enhance immune functions (Dorper et al., 2021). In this respect, Adams and Koutsos (2024) indicated that the nutrient content of BSFL frass makes it appropriate for use in animal feed, since BSFL frass contains lauric acid, chitin, and antimicrobial peptides, which may improve overall animal health. Chitin, a prevalent polysaccharide in insect exoskeletons, may act as a prebiotic, positively impact gut health by modulating microbiota composition, reducing harmful microorganisms, and improving gastrointestinal health and nutrient digestibility (Borrelli et al., 2017), leading to enhanced feed conversion ratios (Bovera et al., 2016).

Furthermore, studies by Moniello et al. (2019) noted that chitin in insect meal positively affects small intestine morphology, brush border enzymatic activity, as well as caecal microbial activity. They explained that chitin induced increases caecal production of volatile fatty acids (**VFAs**) like acetate and butyrate (Cutrignelli et al., 2018), which is considered the prime enterocyte energy source. When a higher amount of butyric acid is available, the increase of nutrients for enterocytes enhances blood flow through the intestine and then tissue oxygenation and nutrient transport and absorption. These signals may induce the production of growth factors able to stimulate the growth of the different intestine tracts, thus to have an impact on the body growth (Mahdavi and Torki, 2009).

On the other hand, the results of our study are likely also due to the positive impact of BS, a probiotic, which combines digestive enzymes (cellulase, hemicellulase, α-amylase, protease), along with non-pathogenic anaerobic live microorganisms (Ruminococcus flavefaciens). When added to chicks' diets, this probiotic improves the utilization of **MFRR** meals and other feed ingredients by enhancing their nutritive value, promoting fiber hydrolysis, increasing fiber digestibility and boosting nutrient absorption, thereby optimizing nutrient utilization. The enzymes in BS are known to boost feed utilization efficiency by increasing the endogenous enzyme activity and supporting the normal gut flora in chick's, which facilitates the hydrolysis of feed nutrients and enhancing the digestion of fibrous materials like chitin, thus enhancing chicks performance. The benefits of exogenous enzymes and ZAD probiotic in enhancing feed utilization, growth, intestinal health, immune response and economic returns are supported and well-documented in literature (Alagawany et al., 2018; Elsagheer et al., 2022). Our findings align with several studies demonstrating the significant positive effect of adding ZAD probiotic on production performance and immunity (Gado et al., 2013; Gado, 2020; Singh et al., 2019).

Regarding slaughtering performance; carcass characteristics and the relative internal organ weights, present results indicated that partially replacing corn and soybean meal by **MFRR** with or without BS, significantly increased carcass yield and the relative weights of the liver, heart, gizzard, spleen, bursa, and cecum length, while decreasing the relative weight of intestine compared to the control (T1) or BS group (T2). These finding showed a promising effect of incorporating **MFRR** meal with or without BS in Gimmizah chicks diet, a result that could pave the way for commercial applications.

Carcass characteristics extremely depend on the nutritional value and chemical composition of the diet (Zuidhof et al., 2003), and the differences in the organ weights could be correlated with carcass weight as mentioned by Elamin (2013), who found a positive correlation between carcass weight and organ weights. Our results are consistent with those of Vasilopoulos et al. (2023) who found significantly increased carcass yield and spleen weight in broilers fed *Tenebrio molitor* (TM) larva. The higher dressing percentage may be attributed to increased body weight gain which is associated with high essential amino acids content in maggot meal (Okah and Onwujiariri, 2012). Similarly, Ballitoc and Sun (2013) reported that a diet containing 2 % TM increased the carcass yield, slaughter weight, eviscerated weight notably heart and gizzard, while reducing abdominal fat weight. Additionally, quail birds fed on the two different insect meals, peach fruit fly (***Bactrocera zonata***; *B. zonata*) and African cotton leafworm (***Spodoptera littoralis***; *S. littoralis*) exhibited higher relative carcass, liver, heart, spleen and bursa weights. The comparably high weight of carcass could be due to high essential amino acid, fat and energy contents of *B. zonata* and *S. littoralis* meals and the increased lymphoid organ weights can be attributed to enhanced immune function in growing quail chicks (Sayed et al., 2019). This explanation is supported by Oddon et al. (2021), who attributed the higher spleen weight in broilers fed BSFL or TM to increased immune system activity, this outcome likely due to chitin and its immunostimulant properties (Gasco et al., 2018). Furthermore, BSFL included as a partial or full replacement for meat and soybean in broiler diets, positively affected carcass traits (Kumar et al., 2022; Schiavone et al., 2017a, 2018, 2019). Similarly, Housefly maggot meal (**HMM**) included at different concentrations in broiler diets resulted in higher eviscerated yield, spleen and bursa percentage with lowered abdominal fat (Elahi et al., 2020a). Our results also support several studies showing higher relative gizzard weight in chickens fed diets containing 5 % BSF (Onsongo et al., 2018), 2 % TM (Ballitoc and Sun, 2013), or 20 % Housefly larva meal (HFM) (Okah and Onwujiariri, 2012) as a replacement for soybean and fish meals.

Among the intestinal tracts, the ceca of chicks fed **MFRR** with or without BS in the present study had a greater length than that of controls or BS group, a finding that aligns with Hussain et al. (2017) who reported that broilers fed 0.3 % *Tenebrio molitor* (TM) larvae meal had greater ceaca length than those broilers fed soybean meal, attributing this effect to the chitin level of TM, which enhances gut health by improving nutrient digestibility and intestinal morphology.

Hematological and biochemical measurements are essential diagnostic tools that offer crucial insights into a bird's health and metabolic status. These tests provide valuable information and detail that contribute to understanding the physiological condition of the birds, which reflects the relationship between diet and health status (Nunes et al., 2018).

The findings of the current study revealed significant increases in red blood cell counts, hemoglobin, hematocrit, total protein, albumin, globulins, cholesterol, triglycerides, thyroxine, urea, and creatinine with no notable differences in AST, ALT and ALP in treated birds compared to controls. However, since the fluctuations in these results remain within the physiological reference range values (Sturkie's, 2021), it is reasonable to conclude that partial replacement with **MFRR** meal does not negatively affect the birds' metabolism or blood parameters, thus confirming the safety of **MFRR** utilization in poultry diets. Likewise, substituting 2 % BSF (El-Kaiaty et al., 2022), 0.3 % and 10 % TM (Benzertiha et al., 2019, 2020; Biasato et al., 2017, respectively) in broiler diets significantly increased blood total protein, cholesterol, and red blood cells. Our results are also in close agreement with findings by Sayed et al. (2019), who observed similar increases in serum total protein, triglycerides, and cholesterol with no changes in ALP, ALT, or AST levels in quails fed insect-based diets. They suggested that higher triglycerides levels might be due to the higher lipid content of insect-containing diets. Also, Attivi et al. (2020) proposed that increased triglycerides levels in BSF-fed broilers could be a result of stimulated hepatic lipogenesis, the triglycerides are used for oxidation to produce more energy, thus the chickens of this group would have more energy for metabolism process and this may also partly explains their better growth performance. Similarly, Elahi et al. (2020a) reported increased red blood cells, packed cell volume, total protein, and uric acid significantly with no significant impact on AST enzyme activity in broilers fed diets containing the housefly maggot meal (**HMM**) at 4 and 8 %, they attributed the increase in total protein to the excellent amino acid profile of **HMM**. Zang et al. (2018) also found higher total protein, albumin, and globulin in broilers fed on diet containing earthworm meal at 1,3 and 5 %. Albumin concentration was significantly higher in birds fed with BSF as a substitute of fish meal, the increment in serum albumin concentration may be due to high content of essential nutrients in insect meal, improving health, nutrients uptake and subsequently live body weight of the chickens (Attivi et al., 2020). Furthermore, serum globulin levels increased in broilers fed 2.5 % and 5 % TM meal inclusion in their diet (Sedgh-Gooya et al., 2021). Also, BSF meal increased globulin level in laying hens (Marono et al., 2017), likely due to presence of chitin, act as a prebiotic (Selaledi et al., 2019).

AST and ALT enzyme activities crucial key indicators of liver function, with elevated levels typically signifying liver injury or necrosis (Hyder et al., 2013). On the other hand, ALP is involved in bone formation and it is considered a marker of skeletal health, bone disease and liver damage (Seyedalmoosavi et al., 2022). In our study, stable and unchanged levels of AST, ALT, and ALP suggests that **MFRR** has no adverse effects on liver or bone health. These results are consistent with earlier findings by Mahmoud et al. (2022); Biasato et al. (2017, 2018a) and Zang et al. (2018), which reported no changes in ALT and ALP levels in birds fed black soldier fly, mealworm, and earthworm meals, respectively.

Serum urea and creatinine are important valuable indicator of kidney functions. Urea levels are positively correlated with dietary protein intake, while creatinine, a byproduct of phosphocreatine breakdown in muscle, serves as a sensitive marker for both protein metabolism and renal function, with its blood levels being influenced by muscle mass, age, physical activity, and diet. Elevated levels of both can indicate kidney dysfunction due to exposure to toxic substances. Significant increases in serum uric acid level were noted in laying hens fed diets with 5 % and 10 % BSF larvae inclusion (Zawisza et al., 2023) and in broilers fed a 30 % BSF inclusion diet (Seyedalmoosavi et al., 2022). These increases may reflect efficient protein utilization from the insect-based diet (Attivi et al., 2020). Moreover, according to Marono et al. (2017), the lowest creatinine levels observed in hens fed insect meal may be related to lower protein utilization in these birds. In any event, despite differences between groups in the present study, both urea and creatinine levels remained within the normal physiological range, indicating that the dietary treatments did not affect renal function.

Regarding thyroxine hormone concentration, the results obtained showed a significant increase in thyroxine concentrations in birds treated with BS or **MFRR**, either alone or combined with BS, compared to controls. This is in accordance with findings by El-Kaiaty et al. (2022), who reported that substitution 4 %, and 6 % black soldier fly increased thyroxine hormone concentration (T4) in broiler chicks compared to controls.

In this study, the histopathological features for the intestine revealed that Gimmizah chicks fed **MFRR** meal (T3) and **MFRR** with BS supplementation (T4) displayed normal histological structure of intestinal villi with wide and tall crypts in between the villi (Fig. 2;c and d, respectively). In contrast, chicks fed on the control diet (T1) and control diet with BS (T2) exhibited histological changes in their small intestine, including infused villi and absence of crypts ((Fig. 2; a and b, respectively). These findings support Weththasinghe et al. (2021), which emphasize the suitability and the positive impact of insect meal on intestinal health and nutrient absorption. In this context, broiler chicks’ intestinal histopathology was unaffected by the dietary inclusion of varying levels of *Tenebrio molitor* (TM) larvae powder at 2.5 % and 5 % (Sedgh-Gooya et al., 2022) or 5 % BSF and TM live larva (Oddon et al., 2021). Vasilopoulos et al. (2024) found that replacing broiler diet with whole dried *Tenebrio molitor* larvae at 5 % and 10 % had no negative impact on intestinal epithelium formation, with the 5 % group showing higher villi in both the duodenum and ileum compared to the 10 % and control groups, indicating a positive effect on the intestinal epithelium, particularly at lower inclusion levels and this aligns with the findings of Sedgh-Gooya et al. (2022). Biasato et al. (2018b), experimented *Tenebrio molitor* (TM) larvae meal at 5 %, 10 %, and 15 % inclusion levels in isonitrogenous and isoenergetic broiler diets. They indicated that a 15 % TM inclusion negatively impacted intestinal morphology, in particular, shorter villi heights (Vh), crypt depth (Cd), and reduced Vh/Cd compared to the 5 % and control diets. This hypothesis further supported by the results of Dabbou et al. (2018), who noted increased villus height (Vh) and crypt depth (Cd) in broilers fed 5 and 10 % BSFM, compared to that of control and those fed 15 % inclusion level, which showed shorter villi (Vh) and higher crypt depth (Cd), signaling poor gut development. Both studies suggest that lower inclusion levels are preferable and more beneficial for gut health, while increasing inclusion levels deeply worsen the gut, as a higher insect content can reduce nutrient digestibility, likely due to the chitin in the insect exoskeleton (Kroeckel et al., 2012; Hossain and Blair, 2007). Several authors have obtained better results by making optimal substitution ratio, recommend partial replacement with insect meal for optimal outcomes (Awoniyi et al., 2003(.

In this regard, the health of gastrointestinal tract (GIT) impacts directly animal productivity. The gut’s role in body health and growth is widely accepted to be expressed through various functions, like pathogen defense, nutrient absorption, immune system maturation and the gut microbiota establishment (Vasilopoulos et al., 2024). The structure of crypts and villi is a very important element of the intestinal epithelium, reflects gut cell proliferation, absorption and its homeostasis (Kwon et al., 2020; Schiavone et al., 2018). Ideally, gut morphology should be characterized by long villi for enhanced digestive enzymes 'operation and nutrient transportation, and shallow crypts that ensure villus longevity (Biasato et al., 2018b). Longer villi and shallower crypts promote the surface area for nutrient absorption, enhancing intestinal cell maturation and digestive enzyme activity (Adeleye et al., 2018).

In the present study, low levels of **MFRR** meal were used, and no negative effects were observed on intestinal morphometric indices. At the same time, it is hypothesized that supplementing with BS aids in converting chitin into the important polysaccharide-based active ingredient ‘chitosan’ which can play a role in shaping the gut, act as an immune-stimulant, anti-inflammatory agent, and reducing intestinal inflammation, and consequently improve the gut health, growth, immunity and decreasing the need of antibiotics in chicks (Wang et al., 2020; Gasco et al., 2018). The biological properties of chitosan, including its biocompatibility, biodegradability, antioxidant, cytoprotective, antimicrobial, and anti-inflammatory activity have been discussed in recent publications (Elnesr et al., 2022). Moreover, broiler chickens fed diets supplemented with 0.6 g of chitosan showed significant improvements in body weight gain, feed intake, and ileal villus area (Khambualai et al., 2009). Supplementation of chitosan in insect-supplemented diet may improve these effects, which needed to be studied in the future.

## Conclusion

One of the main goals of this study was to assess the feasibility of using **MFRR** as a partial substitution for conventional feed ingredients in slow-growing Gimmizah chicks throughout their productive cycle. Under the conditions of this experiment, our findings suggested that replacing 10 % of corn and soybean meal with **MFRR** combined with BS or not, is, viable, and safely used without any adverse effects. The results also propose that the nutrient content of **MFRR** is appropriate for use in poultry feed, and that **MFRR** can be produced at a volume and cost that is feasible and viable for commercial poultry producers. **MFRR** could serve as a valuable and promising alternative in feeding system, positively influencing growth, slaughtering performance, intestinal health and hematobiochemical profile, ultimately effectively contribute to reducing poultry farming costs and bring profitability to poultry industry. This study offers new and valuable insight into the potential use of **MFRR** to partly replace traditional feed ingredients in chicken feeds. Further future studies will be required on the utilization of **MFRR** in poultry feed especially the potential of higher percentages of replacement, the optimal level of substitution and its effects on the meat quality traits. Ethical, legal and safety issues need to be considered, especially if insects rearing residues are used from insects that were not intended as food or feed such as MFRR. In addition, the sustainable impact of the use of MFRR in comparison to alternative utilization pathways such as the use as fertilizer or for biogas production needs to be investigated..

## Disclosures

The authors declare that they have no known competing financial interests or personal relationships that could have appeared to influence the work reported in this paper.
